# Complete Spinal Cord Injury Secondary to Serratia marcescens Spinal Epidural Abscess: A Report of Significant Neurological Improvement After a Delayed Presentation

**DOI:** 10.7759/cureus.44451

**Published:** 2023-08-31

**Authors:** Ashley Gall, Abigail Cowher, John France, Shari Cui

**Affiliations:** 1 Orthopaedic Surgery, West Virginia University School of Medicine, Morgantown, USA; 2 Orthopaedic Surgery, Prisma Health, Columbia, USA

**Keywords:** incomplete spinal cord injury, intravenous drug use (ivdu), decompression of spinal epidural abscess, paralysis, extremely delayed presentation, decompressive laminectomy, serratia marcescens, spinal epidural abscess, american spinal injury association (asia), spinal cord injury

## Abstract

The exact time at which neurological deficits secondary to a spinal cord injury (SCI) become permanent is unknown. However, urgent decompression within 24 hours of insult is advocated to maximize the return of function. Despite previous literature showing poor neurological recovery with intervention after 24-72 hours, multiple cases have since shown noteworthy clinical improvement following significant delays in presentation. We report the case of a 55-year-old incarcerated male who presented to our hospital with a four-week history of a complete (American Spinal Injury Association (ASIA) A) SCI after a prison altercation. The patient exhibited profound deficits of over one-month duration, and magnetic resonance imaging (MRI) revealed an epidural abscess at T7-T8 with severe cord compression and another epidural abscess at L4-L5. This prompted immediate IV antibiotic therapy. A full neurological examination at hospital admission showed a complete absence of sensation, motor, rectal tone, and rectal function below T8, indicating a grade ASIA A SCI. Blood cultures grew *Serratia marcescens*. After thorough deliberation, considering over a month of complete neurological deficits, it was decided that surgical intervention would be unlikely to improve the patient’s clinical status. Nonetheless, after only 24 hours of IV antibiotic administration, the patient progressed from an ASIA A to B, with a return of 100% accurate, although dull, sensation below T8. Within one week, his abscesses diminished on follow-up MRI, yet T7-T8 remained under significant pressure with no further clinical improvements. Due to his unexpected improvement to an ASIA B, which then plateaued at this level, surgery was again discussed in an attempt to maximize recovery. The patient wished to proceed, even given low chances of a meaningful recovery. He subsequently underwent evacuation and decompression. Two weeks postoperatively, the patient advanced from an ASIA B to C; he remained so until discharge 46 days after presentation and 30 days after surgical decompression.

This case is noteworthy within the literature due to two compelling features. Firstly, it represents a significantly delayed presentation of a complete SCI with unexpected, meaningful, and swift improvement after medication and surgical intervention. Secondly, it is one of the few documented cases of *Serratia marcescens* spinal epidural abscess (SEA).

## Introduction

Since the advent of magnetic resonance imaging (MRI) and computed tomography (CT), the diagnosis of spinal epidural abscess (SEA) has become much more efficient, and the mortality rate has decreased from 34% in the 1960s to around 7% today [1]. The mainstay of treatment is IV antibiotics with or without urgent surgical decompression. Time to diagnosis and intervention is the most important factor in avoiding devastating complications, such as paralysis. While surgical decompression performed after 24-72 hours of a complete spinal cord injury (SCI) has traditionally been thought to have a minimal impact on a patient’s neurological recovery, multiple cases have begun emerging in the literature, proving otherwise [2,3]. We report a case of *Serratia marcescens* SEA in which the patient had a complete SCI (American Spinal Injury Association (ASIA) A) of over four weeks duration that improved two ASIA grades after intervention.

## Case presentation

**Figure 1:**
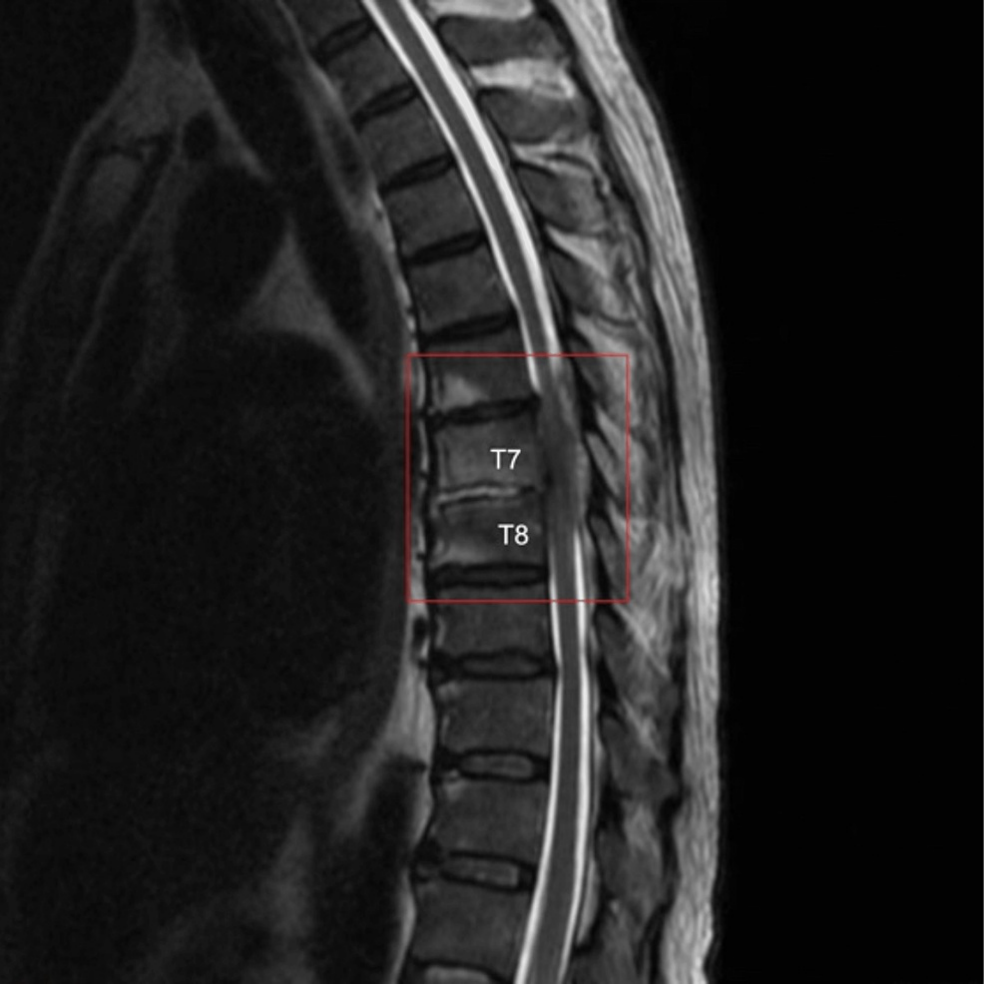
T2-weighted sagittal view revealing T7-T8 discitis/osteomyelitis with associated extensive phlegmonous enhancements and a thin dorsal epidural abscess (red box).

**Figure 2:**
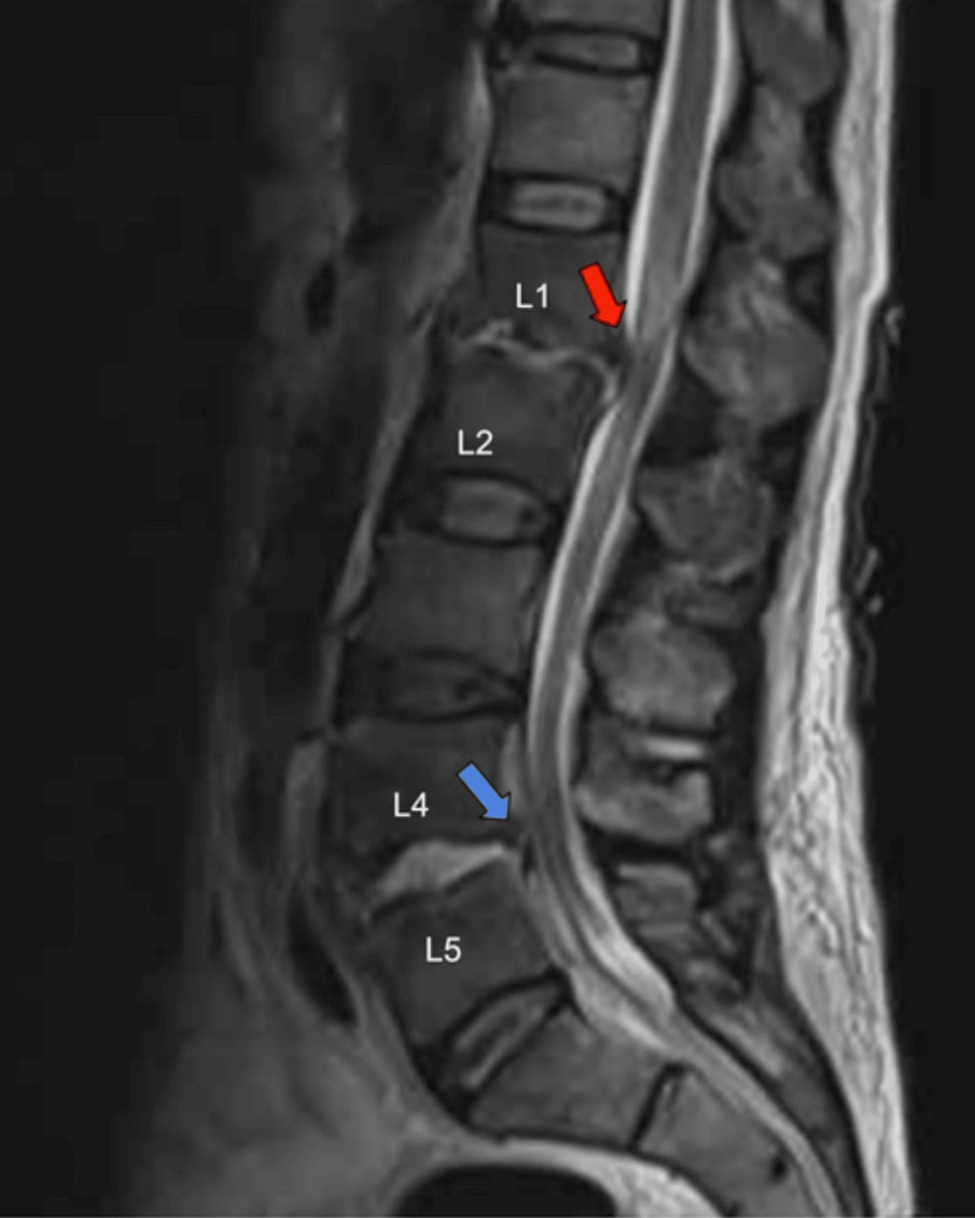
T2-weighted sagittal view revealing L1-L2 and L4-L5 discitis/osteomyelitis with associated extensive phlegmonous enhancements (red arrow) and a thin ventral abscess at L4-L5 (blue arrow).

A 55-year-old incarcerated male with a history of intravenous drug use (IVDU) presented to our hospital with a four-week history of inability to move his lower extremities. He had been involved in an altercation five weeks prior, while incarcerated, during which he was slammed directly onto his back. An immediate “jolt of electricity,” accompanied by pain, was felt throughout his body. Over the following week, he felt progressively weaker until he was unable to ambulate, developed urinary and fecal incontinence, and lost all sensation distal to his umbilicus. Symptoms persisted, and he was brought to the hospital weeks later. Upon physical examination, the patient had 0/5 strength and 0/2 sensation in all respective muscle groups and regions below the level of T8 with flaccid rectal tone and an intact bulbocavernosus reflex. Hence, the injury was classified as an ASIA A at that time [4]. MRI revealed ascending T7-T8, L1-L2, and L4-L5 discitis/osteomyelitis; this was associated with extensive phlegmonous enhancements and effacement of the thoracic spinal cord and cauda equina nerves. A thin dorsal epidural abscess was noted at T7-T8 (Figure 1), and a thin ventral abscess was noted at L4-L5 (Figure 2). Multifocal bilateral psoas abscesses were also present. The patient was immediately started on IV cefepime and vancomycin. Blood cultures grew *Serratia marcescens*, a rare culprit likely introduced during IVDU, and the antibiotic regimen was de-escalated to cefepime only. Of note, the patient’s last IVDU was three months before his admission, just prior to his incarceration.

Given four weeks of paraplegia, surgical decompression was deemed unlikely to allow for the return of any sensation or motor function. Due to the poor clinical prognosis and surgical risks, it was decided to keep the patient on IV antibiotics and conduct a repeat MRI one week later to evaluate epidural abscess improvement. The patient had previously demonstrated a complete absence of sensation on his neurological examination, including being insensate to needle penetration in his legs and feet. However, 24 hours after antibiotic initiation, the patient was able to localize sensation with 100% accuracy at all neurological levels below T8. Given the latter findings, the patient’s SCI grade was re-classified as an ASIA B.

**Figure 3:**
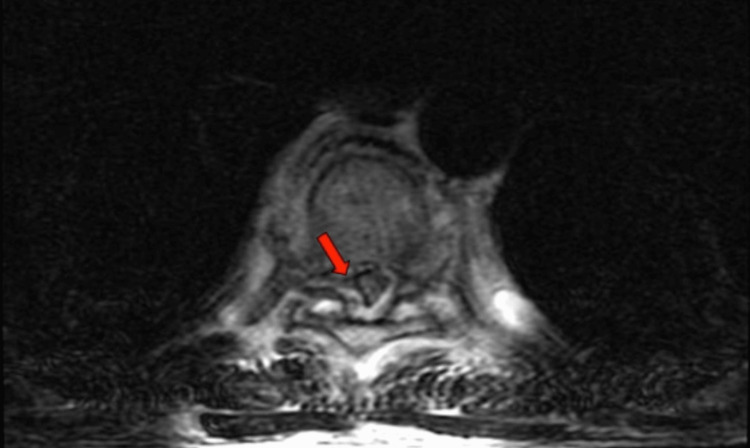
T2-weighted axial view revealing significant compression of the thoracic spinal cord (red arrow).

A surveillance MRI obtained one week later showed a shortening of the abscesses, yet the spinal cord at T7-T8 remained under significant pressure (Figure 3). There was no further meaningful clinical improvement. Due to his unique, sudden advancement to an incomplete SCI, the risk-benefit analysis of surgical intervention was repeated, and it was decided to proceed with surgery. He underwent evacuation of thoracic epidural phlegmon with T6 inferior dome laminectomy, T7 right-sided hemilaminectomy, and T8 left-sided hemilaminectomy. The L4-L5 level was not decompressed due to the small size of the abscess. Two weeks postoperatively, the patient regained the ability to contract his bilateral hip flexors, right tibialis anterior, and right extensor hallucis longus muscle, thereby advancing him from an ASIA B to C.

**Figure 4:**
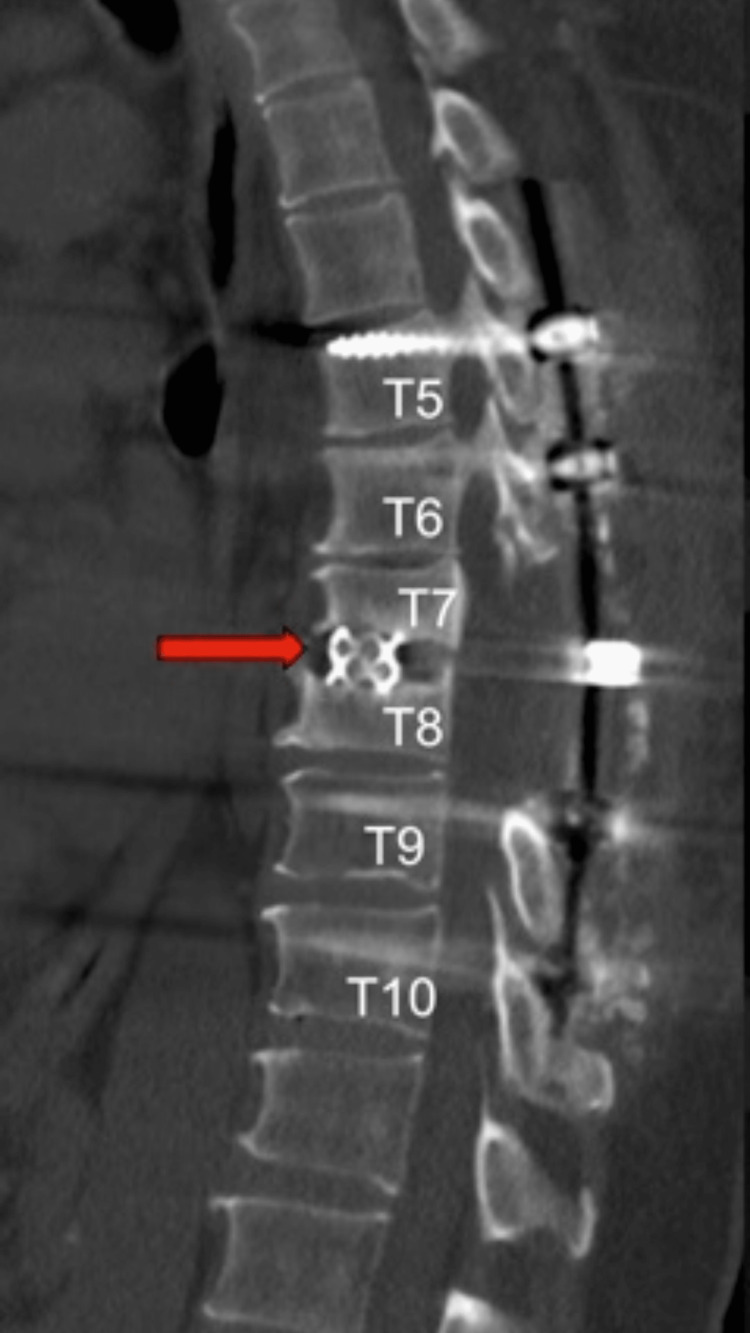
T1-weighted sagittal view after right costotransversectomy and partial anterior corpectomies at T7 and T8 with T7-T8 anterior fusion (red arrow) and posterior T5-T10 fusion.

Approximately one week after discharge, the patient was readmitted due to worsening back pain. Upon physical examination, his SCI remained classified as an ASIA C. MRI showed osteodiscitis at T7-T8 and a progression in the epidural abscess at T6-T9 with cord impingement. Therefore, the patient was again taken to the operating room. The patient received a right costotransversectomy and partial anterior corpectomies at T7 and T8 for debridement with a T7-T8 anterior fusion and a T5-T10 posterior fusion (Figure 4). Operative cultures grew both *Serratia marcescen*s and *Staphylococcus epidermidis*, while repeat blood cultures two days later were negative. The patient was administered a six-week course of IV vancomycin and cefepime, followed by oral levofloxacin. Upon discharge, the patient was able to move his bilateral lower extremities with gravity eliminated (ASIA C). Unfortunately, the patient did not return for any of his follow-up visits.

## Discussion

Over one-third of patients with the diagnosis of SEA will have a poor neurological outcome, and one-quarter will face irreversible paralysis [5-7]. Early diagnosis is a key factor in preventing neurological deficits, yet the presentation of the disease is vague. The “classic triad” of neck and back pain, pyrexia, and neurological impairments is often not found, with only 8% of cases presenting with the triad in a study of 101 patients [1]. A high degree of suspicion must be used as neurological deterioration can occur rapidly, with weakness advancing to paraplegia within 24 hours [8]. After diagnosis, the decision must be made to treat medically alone or to involve surgery.

Medical versus surgical management of SEA has been a long-debated topic with many factors to take into consideration. A 2014 literature review of 28 case series, containing at least 30 patients each, defined which patients may be eligible for exclusive medical management with antibiotics and close monitoring [9]. These patients included those who were unable to undergo an operation, had a complete SCI of over 48 hours in duration with minimal radiographic concern for an ascending lesion, and were neurologically stable without risk factors for medical management failure [9]. Risk factors included age > 65-80, diabetes, cancer, IVDU, smoking, white blood cell (WBC) > 12.5 × 10^9^/L, C-reactive protein (CRP) > 115 mg/L, positive blood cultures, and/or cord compression on MRI or CT [9]. Despite our patient’s history of IVDU, WBC of 15.3 × 10^9^/L, positive blood cultures for *Serratia marcescens*, and MRI showing cord compression, it was decided to treat the patient with IV antibiotics alone because of surgical risks, combined with a poor prognosis for neurological recovery. In spite of a failure rate as high as 40%, medical management worked unexpectedly well for our patient, with a full ASIA grade recovery in all dermatology below the injury level [10,11].

Meaningful functional recovery has been found, even in delayed presentations of complete paralysis, when every case underwent surgical decompression [2]. In a study inclusive of 23 patients with a primary pyogenic SEA, 19 patients were paraplegic for a duration of 4.1 ± 4.1 days prior to laminectomy with the evacuation of an abscess [2]. After a mean follow-up of 4.2 ± 4.3 months, only eight patients remained paraplegic, and one notable patient improved from a Nurick grade 5 to 2. Similarly, our patient recovered with surgical decompression. Another study retrospectively reviewed 128 cases of SEA and found that medical management was more likely to be used in patients with higher average pretreatment motor scores [12]. However, those medically managed patients had significantly less return of motor function compared to those patients with lower pretreatment motor scores who were surgically managed. Inclusion criteria did not specify complete or incomplete SCI status. A review of 1,099 patients found that those presenting without neurological deficits, independent of back pain symptoms, were significantly more likely to undergo medical management compared to those who presented with radiculopathy, paresis, and paralysis [13]. However, the time from the onset of deficits to presentation was not recorded [12]. In sum, the literature lacks clarity in identifying the ideal management for delayed presentations of a complete SCI secondary to SEA.

When considering our patient’s rapid improvement, it is important to examine the interventions utilized and the infection’s source. The patient’s SEA likely originated from hematogenous dissemination secondary to IVDU. In a prospective observational study of 102 patients, individuals with SEA secondary to IVDU had a higher rate of motor score improvement with the surgical intervention compared to non-IVDU [14]. Also, in cases of SEA secondary to IVDU, neurological morbidity may not only be due to neural compression but also ischemia resulting from profound inflammation and disruption of the microvasculature adjacent to the abscess [14]. In this case, blood cultures grew* Serratia marcescens*. This pathogen is known to produce a pore-forming hemolysin called ShIA, associated with the mass release of inflammatory mediators. The gram-negative bacillus *Serratia marcescens* is typically a nosocomial infection following surgical intervention and is rarely a cause of a SEA. The most common causative agent for a SEA is *Staphylococcus aureus*. However, illicit drug use is a risk factor for community-acquired *Serratia marcescens *[15]. Proper IV antibiotic therapy may have swiftly reduced our patient’s inflammation, leading to rapid clinical improvement. Further studies are needed to evaluate why patients with SEA secondary to IVDU may gain back more neurological function than other patients.

## Conclusions

Cases of delayed-presenting complete SCI are traditionally thought to have a very poor prognosis, with neurological recovery highly unlikely. Therefore, such cases tend to undergo less aggressive management. In this article, we present the case of a delayed presentation of a complete SCI secondary to *Serratia marcescens* SEA. The patient did unexpectedly well within a short time period after both medical and surgical intervention, recovering from an ASIA A to C by discharge. Although it is standard of care to rapidly intervene surgically on incomplete spinal cord injuries, this case supports a more aggressive approach to those with complete spinal cord injuries, even with prolonged neurological deficits.
